# From chip-in-a-lab to lab-on-a-chip: a portable Coulter counter using a modular platform

**DOI:** 10.1038/s41378-018-0034-1

**Published:** 2018-11-19

**Authors:** Stefan Dekker, Pelin Kubra Isgor, Tobias Feijten, Loes I. Segerink, Mathieu Odijk

**Affiliations:** BIOS Lab on Chip Group, MESA+ Institute for Nanotechnology, University of Twente, 7500 AE Enschede, Netherlands

## Abstract

The field of microfluidics has been struggling to obtain widespread market penetration. In order to overcome this struggle, a standardized and modular platform is introduced and applied. By providing easy-to-fabricate modular building blocks which are compatible with mass manufacturing, we decrease the gap from lab-to-fab. These standardized blocks are used in combination with an application-specific fluidic circuit board. On this board, electrical and fluidic connections are demonstrated by implementing an alternating current Coulter counter. This multipurpose building block is reusable in many applications. In this study, it identifies and counts 6 and 11 μm beads. The system is kept in a credit card-sized footprint, as a result of in-house-developed electronics and standardized building blocks. We believe that this easy-to-fabricate, credit card-sized, modular, and standardized prototype brings us closer to clinical and veterinary applications, because it provides an essential stepping stone to fully integrated point -of -care devices.

## Microfluidics: standardized and modular devices

A coulter counter based on a standardized modular platform using microfluidic building blocks provides the opportunity for low-cost and portable microfluidic devices. A drawback of current microfluidic devices is that they often have a large physical footprints, and make use of multiple non-standardized components. Now, a team led by Mathieu Odijk at University of Twente in the Netherlands demonstrates a credit card-sized microfluidic device produced from modular building blocks on a fluidic circuit board, providing a standardized and fixed-footprint platform for transferring microfluidics from lab to industry. Moreover, reusable and standardized parts reduce the time for device design and fabrication. As proof of concept they demonstrate an alternating current coulter counter for micron-sized beads.

## Introduction

**Fig. 1:**
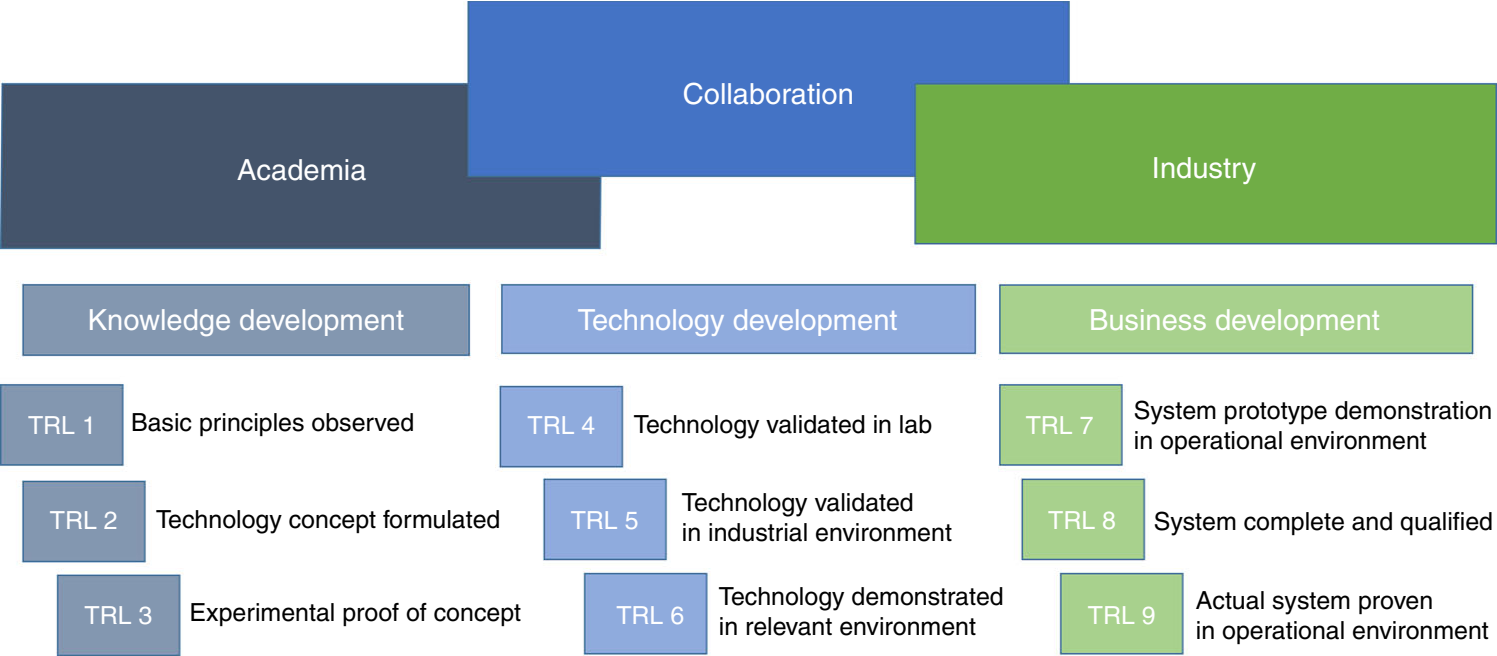
Different stages of innovation. Diagram according to Centre for Process Innovation UK and TRLs according to Horizon 2020

After the initial hype in microfluidics almost three decades ago, which occurred after the introduction of the micro total analysis systems, the field of microfluidics continues to struggle with obtaining widespread market penetration^1,2^. Lack of standards, focus, and communication between academics and industry could be the reason for this struggle^3^. Although many lab-on-a-chip devices are presented in the literature, the term chip-in-a-lab fits better with prototypes currently demonstrated in the microfluidic field^4^. Even though there are a few frontier commercialized devices (Abbott i-STAT and DNA PCR machines), most lab-on-a-chip devices are still stuck at a technology readiness level (TRL) of 3 or 4. Figure 1 shows a diagram with different stages of innovation and their corresponding TRLs^5,6^. If these early prototype devices would become available commercially, they could serve several niche markets and start to decrease the discrepancy between academic output and actual market revenue.

Currently, most of the microfluidic systems are not compact, due to issues with system integration^7^. Even though auxiliary equipment is part of microfluidic set-ups, it is originally intended for other fields, it is bulky, and not easy to miniaturize^8^. Moreover, components for fluid control, visualization, and signal detection are not interoperable with each other, thus commercialization is prevented^7,9^. In order to realize true point-of-care (POC) microfluidic devices, this bulky auxiliary equipment should be miniaturized. Decreasing and standardizing the footprint of auxiliary components will be a step in enhancing portability and reducing equipment dependability. Furthermore, it will improve system integration and compactness, ultimately preventing microfluidic systems to end up in the valley of death between academia and industry.

Until now standards have not been established in the microfluidic field, which leads to adoption of equipment from other fields, for example, smart phones for read-out^10^, microscope slides, microtiter plates, and Luer connectors^11^. Therefore, it is difficult to assemble them in one compact system, which often manifest itself as a so-called spider web assembly in the lab where many tubes and wires connect the various parts to each other. Moreover, the microfluidic community prefers a wide variety of substrate materials, including glass, silicon, and polymers, such as polydimethylsiloxane (PDMS). Even though PDMS is a popular material amongst researchers, it lacks high volume and low cost fabrication and does not have long shelf life, making it unsuitable for realizing commercial products^8,12,13^. In order to commercially benefit from already presented microfluidic devices in academia, which are based on a wide variety of materials, a standardized platform is needed. To provide successful system integration, a large consortium of major industrial and academic partners have introduced standardized elements that maintain compatibility and interoperability between various system parts. Most of the standards that are described in the ISO workshop agreement 23:2016^14^ and whitepapers^15,16^ relate to footprint and interconnect positions of smaller microfluidic elements. In this study, microfluidic building blocks (MFBBs) with a standard footprint are prepared according to ISO workshop agreement and assembled on a fluidic circuit board (FCB). The modular platform combines multiple materials, including a glass Coulter counter chip, plastic MFBBs, and an FCB that is fabricated using rapid prototyping. Instead of PDMS, using a polymer like cyclic-olefin copolymer (COC) provides high volume manufacturing while maintaining biocompatibility and Food and Drug Administration (FDA) approval.

In this paper, we reduce the gap between academia and industry one step closer by demonstrating the implementation of a commonly used technology on a standardized platform. The widely applicable alternating current (AC) Coulter counter is chosen, since it supports label-free detection of single cells, detection of morphological defects and categorization of different cell types^17^. The possibility of performing cell sorting, cancer research, and drug screening makes an AC Coulter counter a favorable building block^18,19^. In this study, the AC Coulter counter previously demonstrated by Segerink et al.^20^ is combined with already available building blocks^21^. The resulting credit card-sized, modular system provides label-free, real-time detection of different sized beads. Elimination of auxiliary components is not yet possible. However, an in-house developed, portable, and low cost lock-in amplifier based on a digital signal processor (DSP) evaluation board with custom front-end increases the possibility of having it in a MFBB format in the future. The standardized MFBBs facilitate easy transition of this prototype to the market.

## Results and discussion

### Fabrication results

**Fig. 2:**
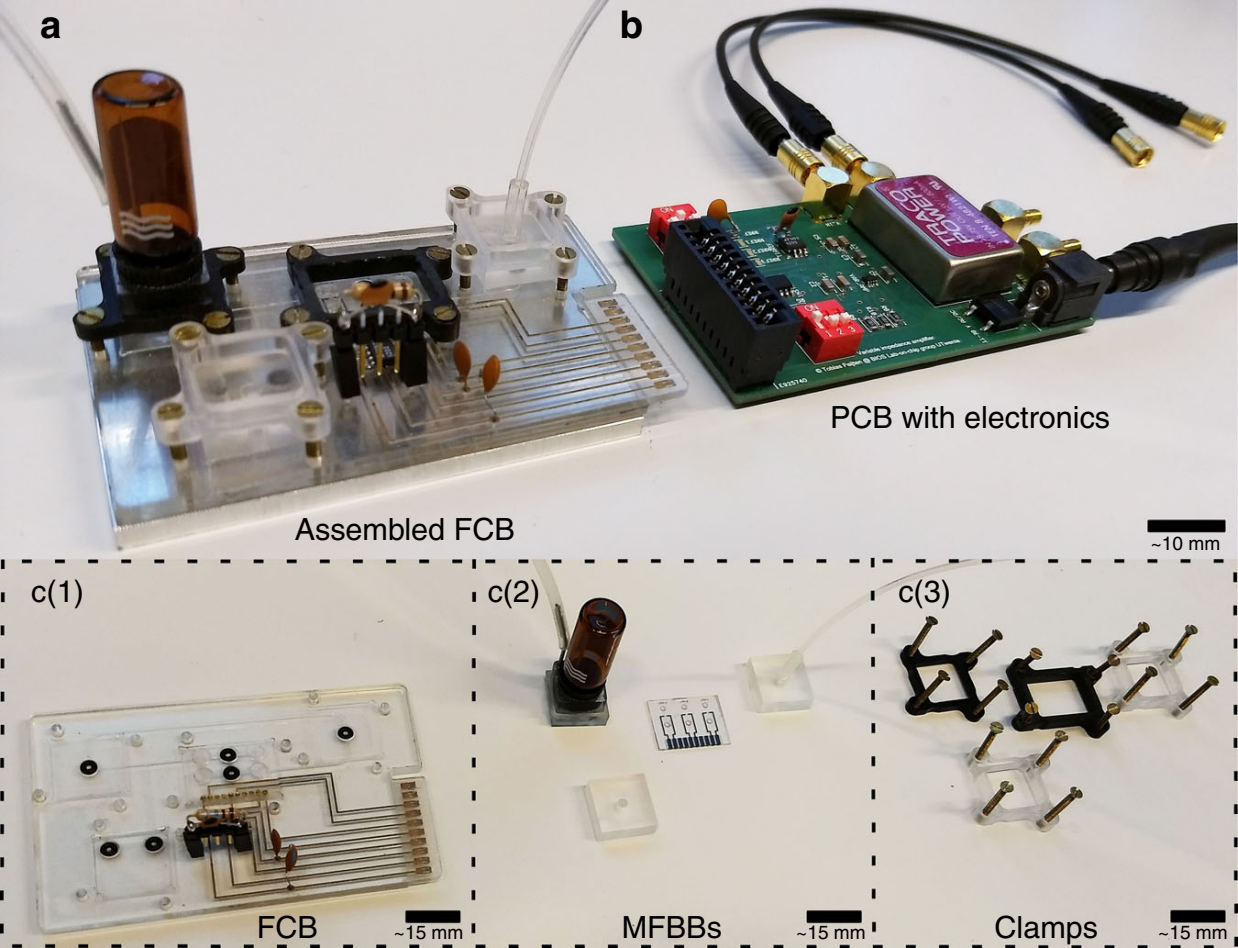
Portable Coulter counter modular platform. **a** Assembled FCB for the particle counting. **b** Electronics for the particle counting. **c** Fluidic parts of the system: (1) FCB with integrated electronics, (2) MFBBs, and (3) clamps to assemble the system, respectively

Owing to utilization of the standard described in ISO Workshop agreement 23:2016^14^, individual MFBBs that provide various functions are assembled onto a credit card-sized FCB (Fig. 2a, c(1)). The printed circuit board (PCB) holds all necessary electronics needed for the pseudo-differential measurement and increases the compactness of the system (Fig. 2b). Interfacing and assembling MFBBs (Fig. 2c(2)) onto the FCB (Fig. 2c(1)) provides functionality and interconnection, respectively. Necessary tools for assembly are shown in Fig. 2c(3). These standardized parts can be put in an online repository to be easily integrated in future projects^22^. The standardization process together with MFBBs facilitates increasing the TRL (Fig. 1) and decreases the gap between the “chip-in-a-lab” concept and a commercial POC device.

### Experimental results

**Fig. 3:**
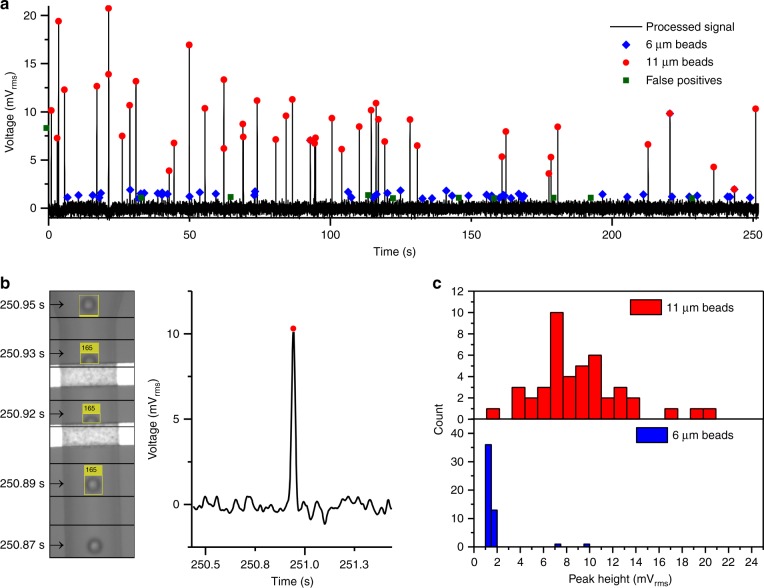
Measurement results from the Coulter counter platform. **a** Differential impedance measurement with 6 and 11 μm beads. In total 101 beads were electrically detected, 46 of them are 6 μm, 46 of them are 11 μm beads, and 9 of them are false positives. **b** Impedance data of an individual 11 μm bead combined with the video frames used to obtain its trajectory. **c** Histograms of the peak amplitudes for a mixed solution

The AC Coulter counter chip integrated onto an FCB is used to detect 6 and 11 μm polystyrene beads in phosphate-buffered saline (PBS). Three sets of experiments are conducted. Solutions that contain only 6 μm beads, only 11 μm beads, or both of them in 1:1 (v/v) ratio are pumped through the microfluidic channels. The results for these measurements are shown in Table 1. The video data are used to obtain the trajectory of the bead as well as the size of a bead, which is subsequently linked to the electrical signal. An exemplary result of this process is shown in Fig. 3b. Also, a video showing this process can be found in the Supplementary Material (Video S1). As Table 1 shows 6 μm beads were detected while running the 11 μm bead solution. Although the system in this case should only detect 11 μm beads as only those kind of beads are present in the bulk solution, some 6 μm beads are still detected from earlier experiments that are left behind in the channels. In order to calculate the detection rate, the number of video detection matched to an electrical signal peak is divided by the total amount of beads detected in the video.

**Table 1:** Number of detected beads in the datasets: video, electrical, and combined

Exp.	Video data No. of beads		Electrical data No. of peaks	Combined No. of video detections matched with electrical peaks					Average bead velocity
	6 μm	11 μm		6 μm		11 μm		False positives	
				No.	Detection rate	No.	Detection rate	No.	
6 μm solution	173	0	164	148	86%	0		14	110 μm/s
11 μm solution	84	85	113	27	32%	84	99%	3	344 μm/s
Mixed solution	118	46	101	46	39%	46	100%	9	289 μm/s

The results show that both solutions that contain 11 μm beads have a detection rate of more than 99%. The detection of 6 μm beads was more challenging, since the peaks generated are close to the noise band (Fig. 3a). One mV_rms_ was deemed to be a suitable threshold for peak detection, since the root mean square (RMS) level of the noise was 0.307 mV_rms_. The largest contributor to the noise was the minimal shielding on the FCB. Even though the threshold was set to three times of the RMS level of the noise, some false positives still occurred. The threshold was kept fixed to prevent lowering the detection rate. This threshold resulted in detection rates of 86, 39, and 32% for 6 μm beads at velocities of 110, 289, and 344 μm/s, respectively. From these results it is clear that detection rate was negatively influenced by higher flow speeds, since higher flow speeds result in higher frequency components in the detected peaks. Depending on the filter setting in the lock-in amplifier, removal of these high-frequency components leads to a peak amplitude below the threshold for 6 μm beads. Increasing the bandwidth of the filters to allow the higher frequency components to pass is not favorable as the noise would also increase.

**Fig. 4:**
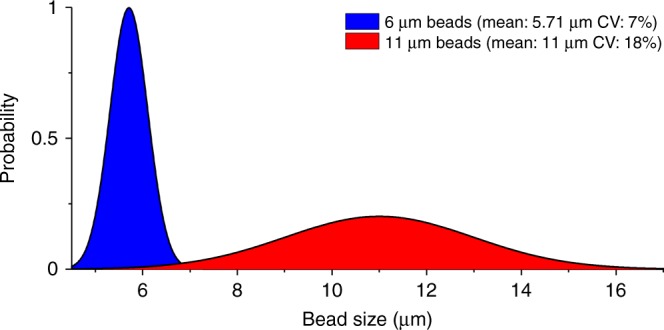
Size distribution (based on a normal distribution and mean/CV figures of the manufacturer) of beads in mixed solution, showing a slight overlap around 7 μm

The distribution of detected peak amplitudes for the mixed solution is shown in Fig. 3c. The peak amplitudes for the 11 μm beads are between 1.9 and 20.7 mV_rms_, while the majority of peak amplitudes for 6 μm beads is between 1 and 1.8 mV_rms_, with an average of 1.6 mV_rms_. Since the total amplification of the pseudo-differential measurement electronics is 100 kV/A, it results in a 16 nA current difference between the resistor-capacitor (RC) reference and the microfluidic chip. A 6 μm bead passing the electrodes generates a difference of 0.3% in the total current flowing between the electrodes. The wide distribution of the peak amplitudes for 11 μm beads (Fig. 3c) can be explained by size variation of the beads, as well as their position in the non-uniform electric field between the planar electrodes. The coefficients of variation (CV) supplied by the manufacturer are 18 and 7% for the 11 μm and 6 μm beads, respectively (Fig. 4). This variation results in a small overlap of the populations. Due to this overlap, both populations can generate equal peak amplitudes. Moreover, the peak amplitude of the signal depends on bead volume instead of bead diameter. Therefore, the histogram in Fig. 3c has a broader distribution than the plot in Fig. 4. The two outliers in the 6 μm peak amplitude histogram are 6 μm beads, which were falsely linked to an electrical peak of a 11 μm bead. This occurs when a 6 μm bead is not electrically detected and a 11 μm bead is present in the same 100 ms time interval.

In order to detect particles smaller than 6 μm, a reduction of the noise level is needed. This can be realized by better shielding of the FCB. The presented system could be used for the detection and counting of cells with diameters in the range of 10 to 15 μm like white blood cells. Improvement in the noise level will enable bacteria and yeast cell detection which have sizes in the range of 1 to 6 μm.

## Materials and methods

### Coulter counter chip

**Fig. 5:**
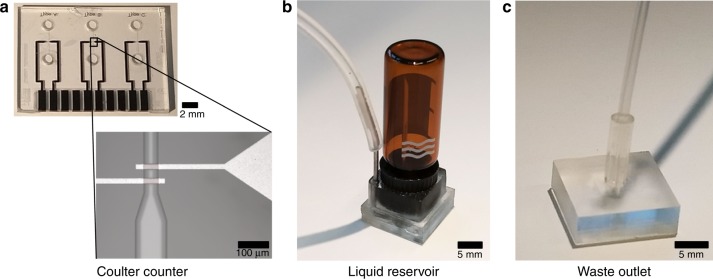
Modular building blocks used in the platform. **a** Coulter counter chip with a detailed view of coplanar electrode pair used for detection. **b** Inlet reservoir MFBB for bead solution. **c** Outlet reservoir MFBB for waste disposal

In this study, we combined the Coulter counter chip that is demonstrated by Segerink et al.^20^ with our standardized platform in order to pave the way for a POC device. This chip consists of coplanar electrodes that are fabricated in a microfluidic channel, as shown in Fig. 5a. An in-house developed lock-in amplifier is used to apply an alternating potential across the electrodes, resulting in an electric field inside the fluidic channel. The impedance of the system increases approximately 0.01%, when an insulating polystyrene bead passes through this electric field.

### Design of the microfluidic system

A design philosophy was used where standardization plays a major role. The system consists of standardized MFBBs that provide the functionality of the system along with aforementioned Coulter counter chip. An FCB is designed to connect these functional blocks. Interconnect positions between the MFBBs and the FCB are standardized. A footprint and interconnect grid for the MFBB are described in the design guidelines^14–16^. Owing to this standardization approach, the MFBBs are reusable. Figure 5a–c show the MFBBs used to assemble the microfluidic system: a Coulter counter chip (Fig. 5a), a 1.5 mL reservoir containing the sample (Fig. 5b) and a polymethyl methacrylate (PMMA) block to connect tubing to a waste reservoir (Fig. 5c). An external pressure regulator (Fluigent MFCS-4C, France) is used to pump the bead solution through the system. Electronics is developed in-house to amplify and record the impedance signals generated in the Coulter counter chip.

### Detection and signal processing electronics

**Fig. 6:**
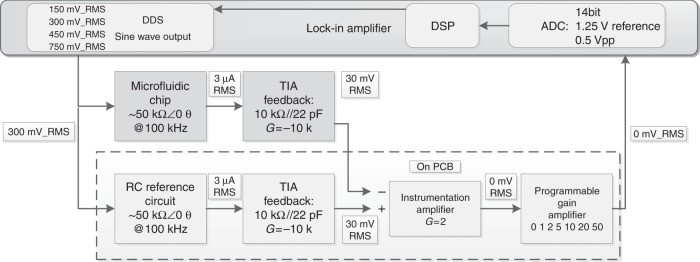
Block diagram illustrating the pseudo-differential measurement. Parts within the dotted line are mounted on the PCB

In order to detect the impedance signals that the Coulter counter chip generates, a two-step solution is used. As stated before, only very small changes in impedance of approximately 0.01% generated by the passing beads needs to be detected. Sensitive electronics were developed, including a (pseudo-) differential measurement for detection. Figure 6 shows a block diagram of this pseudo-differential setup. A resistor and capacitor reference circuit was used to mimic the behavior of the Coulter counter chip. As a result, the small changes in impedance are recorded with more detailed in the dynamic range of the 14-bit analog-to-digital converter (ADC) of the lock-in amplifier. When a bead passes by the electrodes, a difference in current through the Coulter counter chip arises. The signal of interest is obtained by subtracting the Coulter counter signal from the reference signal. The programmable gain amplifier matches this signal to the dynamic range of the input for the next stage. Additionally, an in-house-developed digital lock-in amplifier is used to significantly reduce the amount of noise in the recorded signal. Moreover, the trans-impedance amplifier was placed close to the Coulter counter chip on the FCB to prevent issues with noise. All other electronics are soldered on a PCB.

The in-house-developed lock-in amplifier consists of three main parts: an ADC, a DSP, and a direct digital synthesis (DDS) integrated circuit (Fig. 6). Both the ADC and DDS provide an interface between the analog domain and the digital domain. The DDS generates a sinusoidal signal that is applied to the Coulter counter chip and the analog output signal of it is digitized by ADC. Moreover, the lock-in process is performed by a DSP to prevent additional noise.

In this study, we used three MFBBs: a detection unit (Coulter counter chip), an inlet reservoir, and an outlet reservoir. The inlet reservoir MFBB is a gas-tight 1.5-mL high-performance liquid chromatography vial which is connected to two syringe tips in a PMMA block with a standard footprint^21^. The Coulter counter chip is fabricated out of two glass substrates, as described previously^20^. In the first glass layer microfluidic channels are etched, and fluidic inlet holes are powder blasted. On the second glass layer platinum electrodes are patterned by a recess etch, sputtering, and lift-off process. These two layers are fusion bonded together^20^. The outlet reservoir MFBB is a PMMA block with a standard footprint. A hole (3 mm) is drilled to place Tygon tubing fitted around 1/16 in. Teflon tubing.

**Fig. 7:**
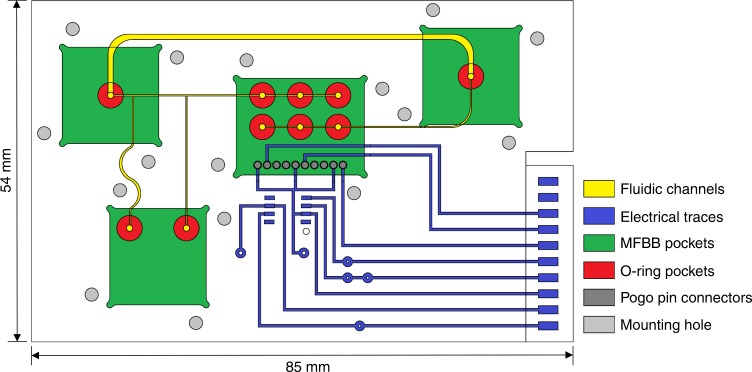
Layout of the FCB providing fluidic and electrical connections between different microfluidic building blocks

The FCB is fabricated using two plates of 2-mm-thick COC (DENZ BIO-Medical, Austria) (Fig. 7). COC is a good candidate for high volume manufacturing, because it can be injection molded and is FDA approved. The top side and bottom side of the top plate are processed for electrical connections and fluidic connections, respectively. Micromilling is used to fabricate fluidic channels and electrical traces. There are two microfluidic channels of different size in the FCB. One of them directs the fluid to the Coulter counter chip (width: 200 μm) and the other one is used for bypassing (width: 800 μm). The depth of both fluidic channels is 200 μm, while the width and height of electronic traces are 400 μm. These dimensions are suitable for using a standard small outline integrated circuit package. For bonding, the surfaces of two COC plates are exposed to cyclohexane vapor for 3 min and afterwards immediately pressed together for 15 min at 2.1 MPa pressure at 110 °C (Carver 3889CEB.4NE1001). Mounting holes are drilled, in order to allow anchoring of MFBBs to the FCB using clamps. The grooves for the electrical traces are filled with silver/silver chloride paste (Gwent C2051014P10) to provide electrical connections. Before the paste cures, electrical components are placed, eliminating the need to solder afterwards.

O-rings between the MFBBs and FCB facilitate a fluidic seal. Pogo pins (Smiths Connectors, 101582) are used to obtain an electrical connection between the FCB and the Coulter counter chip. A trans-impedance amplifier is mounted on the FCB and additional electronic circuitry is provided by a separate PCB, which is connected to the FCB with a card edge connector.

### Experimental

Six micrometer bead solution (Polystyrene Red Dyed Microsphere, Polyscience Europe, Germany) and 11 μm bead solution (Copolymer Microspheres 7510A, Thermo Fisher, USA) with a concentration of 10^6^ beads/mL in PBS (Sigma Aldrich, The Netherlands) are prepared. Tween-20 (Sigma Aldrich, The Netherlands) is added to both solutions with a volume fraction of 0.1% to prevent clustering of the beads. These solutions are thoroughly mixed before they are loaded into the inlet reservoir MFBB. In addition to a height difference of 1 cm between inlet and outlet reservoir, a pressure pump is initially used to introduce fluid into the channel. The current through the Coulter counter chip is recorded at 0.3 V_RMS_, 90 kHz in real time using a LabView program interfacing the lock-in amplifier. The total amplification of the electronics is 100 kV/A.

The recorded data are subsequently processed with a MATLAB (MathWorks, USA) script, in order to detect peaks that represent beads. A moving average filter is applied to the signal and the result of this operation is subtracted from the original signal to remove drift in the signal. Peak amplitudes and corresponding times are obtained from this processed signal and compared to the video data.

Video data are obtained during the experiment, using a microscope (Leica DM LM and SUSS MicroTec, Germany) and a high speed camera at 60 fps (Grasshopper 3, Point Grey, USA). A MATLAB script is used to analyze the resulting video. The script detects the amount of pixels the bead occupies, in order to identify different bead sizes. The location and size information for each bead is saved to determine the exact time that the bead passes the electrodes. Subsequently, this time is used to find the corresponding peak in the impedance data. Overall this script provides information about detected bead size, peak amplitude, and bead velocity for each bead.

## Conclusion

In this paper, we demonstrate a low-cost, modular particle counting microfluidic system with a credit card-sized footprint. The reusable standardized parts decrease the design and fabrication time, which leads to a reduction in the time-to-market for industrial development. The standard platform provides the possibility of integrating different materials in one system. Here, a COC, a PMMA, and a glass device are combined. The low cost of in-house built electronics and lock-in amplifier facilitates a route to commercialization while still keeping system performance up to par. We achieve to measure a 0.3% change in the impedance signal using a pseudo-differential measurement. As a result, 6 and 11 μm polystyrene beads were differentiated. All 11 μm beads were electrically detected, while the detection rates for 6 μm beads were 86, 39 and 32% depending mostly on the flow rate used. Better control over this flow rate will ensure a higher detection rate in the future. Moreover, better shielding of especially the trans-impedance amplifier on the FCB will reduce the noise levels. Most importantly, we show that this standardized platform bridges the gap from “chip-in-a-lab” to “lab-on-a-chip”. The only drawback of the system is the use of an external lock-in amplifier and a pressure regulator. However, we are confident that we can shrink the size of these two components towards obtaining a commercial product.

## Electronic supplementary material

Video S1

Supporting information + figures

## References

[1] Manz A, Graber N, Widmer HM (1990). Miniaturized total chemical analysis systems: a novel concept for chemical sensing. Sens. Actuators B Chem..

[2] Becker H (2009). Hype, hope and hubris: the quest for the killer application in microfluidics. Lab Chip.

[3] Salgado, G. *Barriers to the Diffusion of Microfluidics from Research to Market* (Catolica Lisbon, 2016). Available at: https://repositorio.ucp.pt/bitstream/10400.14/20349/1/Master-Thesis-Gon%C3%A7alo-Salgado.pdf. Accessed 9 Sept 2018.

[4] Neužil P (2014). From chip-in-a-lab to lab-on-a-chip: towards a single handheld electronic system for multiple application-specific lab-on-a-chip (ASLOC). Lab. Chip..

[5] The Innovation Phase—CPI. Available at: https://www.uk-cpi.com/about/the-innovation-phase. Accessed 4 Apr 2018.

[6] Technology readiness levels. Available at: http://ec.europa.eu/research/participants/data/ref/h2020/wp/2014_2015/annexes/h2020-wp1415-annex-g-trl_en.pdf. Accessed 15 Mar 2018.

[7] Volpatti LR, Yetisen AK (2014). Commercialization of microfluidic devices. Trends Biotechnol..

[8] Mohammed MI, Haswell S, Gibson I (2015). Lab-on-a-chip or Chip-in-a-lab: challenges of commercialization lost in translation. Procedia Technol..

[9] Chin CD, Linder V, Sia SK (2012). Commercialization of microfluidic point-of-care diagnostic devices. Lab Chip.

[10] Gubala V, Harris LF, Ricco AJ, Tan MX, Williams DE (2012). Point of care diagnostics: status and future. Anal. Chem..

[11] van Heeren H (2012). Standards for connecting microfluidic devices?. Lab Chip.

[12] Temiz Y, Lovchik RD, Kaigala GV, Delamarche E (2014). Lab-on-a-chip devices: how to close and plug the lab?. Microelectron. Eng..

[13] Mukhopadhyay R (2007). When PDMS isn’t the best. Anal. Chem..

[14] *IWA 23*:*2016—Interoperability of microfluidic devices—Guidelines for pitch spacing dimensions and initial device classification*. Available at: https://www.iso.org/standard/70603.html. Accessed 15 Mar 2018.

[15] van Heeren, H. et al. *Design Guideline for Microfluidic Device and Component Interfaces (part 1) ver. 3*. (2016). 10.13140/RG.2.1.1698.5206 Available at: http://mf-manufacturing.eu/wp-content/uploads/Design-for-Microfluidic-Interfacing-White-Paper-part-1-version-3.pdf. Accessed 15 Mar 2018.

[16] van Heeren, H. et al. *Design Guideline for Microfluidic Device and Component Interfaces (Part 2) ver. 3*. (2016). 10.13140/RG.2.1.3318.9364 Available at: http://mf-manufacturing.eu/wp-content/uploads/Design-for-Microfluidic-Interfacing-White-Paper-part-2-version-3.0-1.pdf. Accessed 15 Mar 2018.

[17] Sun T, Morgan H (2010). Single-cell microfluidic impedance cytometry: a review. Microfluid. Nanofluidics.

[18] Yang RJ, Fu LM, Hou HH (2018). Review and perspectives on microfluidic flow cytometers. Sens. Actuators B Chem..

[19] Xu Y (2016). A review of impedance measurements of whole cells. Biosens. Bioelectron..

[20] Segerink LI, Sprenkels AJ, ter Braak PM, Vermes I, van den Berg A (2010). On-chip determination of spermatozoa concentration using electrical impedance measurements. Lab Chip.

[21] Dekker S (2018). Standardized and modular microfluidic platform for fast Lab on Chip system development. Sens. Actuators B Chem..

[22] MicroFluidics Manufacturing project website. Available at: http://www.makefluidics.com/. Accessed 15 Mar 2017.

