# Supplementary material for: From chip-in-a-lab to lab-on-a-chip: a portable Coulter counter using a modular platform
Source: Microsyst Nanoeng. 2018 Nov 19;4:34. doi: 10.1038/s41378-018-0034-1 (PMC6240576; doi:10.1038/s41378-018-0034-1)
Supplement: Supplementary file 2 — Supporting information + figures [file 41378_2018_34_MOESM2_ESM.pdf]

Supporting information

From chip-in-a-lab to lab-on-a-chip: a portable coulter counter using a modular platform

Stefan Dekker, Pelin Kubra Isgor, Tobias Feijten, Loes I. Segerink, and Mathieu Odijk*

*corresponding author (email: m.odijk@utwente.nl)

S1. Video of electrode area and electrical signal

The video shows part (from 100 seconds to 130 seconds) of the experiment shown in Fig. 6. The video is slowed down 30x to improve visibility of the beads. The electrical signal that was taken during the experiment was shown. As beads pass by the electrodes the resulting peak in the electrical signal can be observed.

S2. Supplementary data from video and electrical data processing

Fig. S2 shows the data that was obtained by processing video and electrical data. Fig. S1.a shows the bead trajectories while they passed by the electrodes. Fig S2.b shows the dependence of the peak amplitude on bead speed. Fig. S2.c demonstrates a parabolic velocity profile in the channel. Fig. S2.d shows the dependence of the peak amplitude on bead position in the channel. It also shows that the 11 µm beads are positioned toward the centre of the channel. Fig. S2.e shows the dependence of the peak amplitude on bead size that was detected by the video processing algorithm.

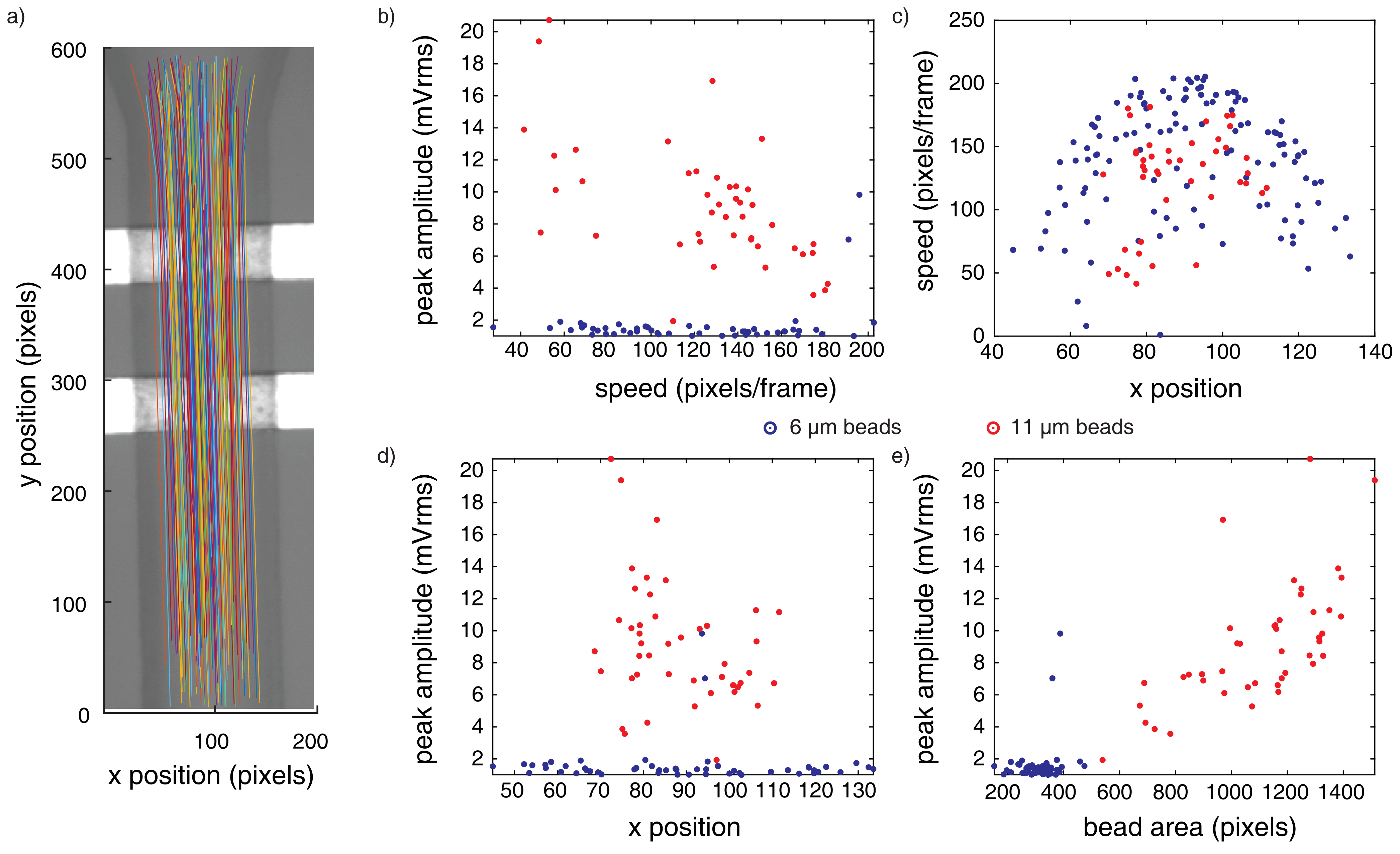

Figure S2 - a) Trajectories of beads overlayed on an photograph of the electrode area. b) Scatter plot showing relation between bead speed and peak amplitude. c) Fluid velocity profile across the x axis. d) Scatter plot showing relation between bead position and peak amplitude. e) Scatter plot showing relation between bead size (area) and peak amplitude.
